# Accidental finding of a toothpick in the porta hepatis during laparoscopic cholecystectomy: a case report

**DOI:** 10.1186/1752-1947-5-421

**Published:** 2011-08-30

**Authors:** Waleed Al-Khyatt, Farhan Rashid, Syed Y Iftikhar

**Affiliations:** 1 Division of Upper GI Surgery, School of Graduate Entry Medicine and Health, University of Nottingham, Royal Derby Hospital, Uttoxeter Road, Derby, DE22 3DT, UK

## Abstract

**Introduction:**

Unintentional ingestion of a toothpick is not an uncommon event. Often the ingested toothpicks spontaneously pass through the gut without sequelae. However, serious complications can happen when these sharp objects migrate through the gastrointestinal wall.

**Case presentation:**

In the current report, we describe the case of a 37-year-old Caucasian woman with an incidental finding of a toothpick in the porta hepatis during laparoscopic cholecystectomy for symptomatic gall stones.

**Conclusion:**

Toothpick ingestion is not an uncommon event and can predispose patients to serious complications. In this particular case, the toothpick was only discovered at the time of unrelated surgery. Therefore, it was important during surgery to exclude any related or missed injury to the adjacent structures by this sharp object.

## Introduction

Unintentional ingestion of a toothpick is not an uncommon event. Often the ingested toothpicks spontaneously pass through the gut without sequelae [1]. However, serious complications can happen when these sharp objects migrate through the gastrointestinal wall [2]. Patients with ingested toothpicks in the gastrointestinal tract typically have no recollection of the event. Symptoms related to toothpick ingestion are often variable and non-specific [3,4]. In the current report, we describe the case of a 37-year-old Caucasian woman with an incidental finding of a toothpick in the porta hepatis during laparoscopic cholecystectomy for symptomatic gall stones.

## Case presentation

**Figure 1:**
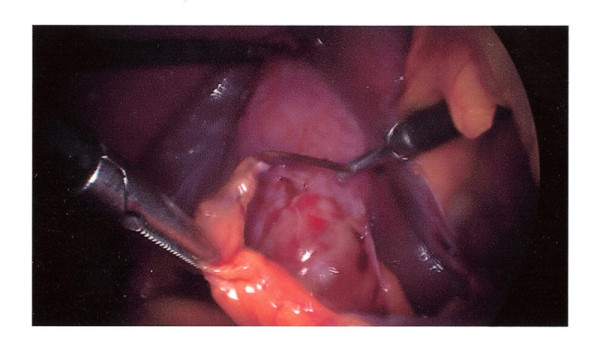
**Toothpick accidentally found in porta hepatis during laparoscopic cholecystectomy**.

**Figure 2:**
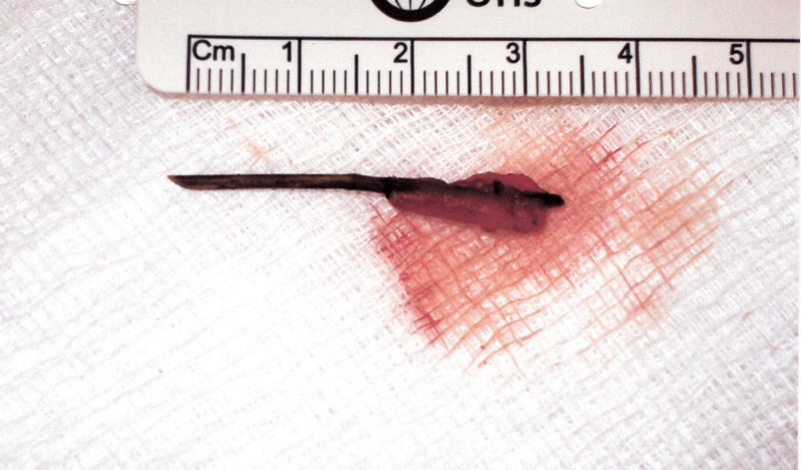
**The toothpick after laparoscopic extraction**.

A 37-year-old Caucasian woman presented to our facility with recurrent attacks of upper abdominal pain over a six-month period. Otherwise, she was fit and well with no significant medical history. The results of general and abdominal examinations were normal. Results from her initial blood tests showed deranged liver function of the obstructive type. An abdominal ultrasound scan revealed a thickened gall bladder wall containing multiple gall stones. A magnetic resonance cholangiopancreatography (MRCP) study showed multiple gall stones; however, there was no choledocholithiasis. Our patient underwent elective laparoscopic cholecystectomy and on-table cholangiogram (OTC) for symptomatic gall stones. The procedure was performed with a standard Veress needle using the pneumoperitoneum technique, with four ports for maintenance of intraperitoneal pressure at 12 mmHg and a pneumoperitoneum time of 55 minutes. During surgery, a foreign body was found wrapped in the omentum and stuck to the liver at the base of the falciform ligament near the porta hepatis (Figure 1). With laparoscopic dissection, this object was removed and revealed to be a foreign body (a toothpick; Figure 2). The duodenum, stomach and hepatic flexure were assessed thoroughly; no evidence of perforation or injury was identified. The laparoscopic cholecystectomy and OTC were performed as planned. Our patient made an uneventful post-operative recovery. During her follow up visit, our patient was informed of this unusual finding. She was able to recall the event of probable accidental ingestion of a toothpick seven years previously. However, she could not remember any significant related illness subsequently.

## Discussion

Foreign body ingestion is a common event which may happen accidentally or intentionally [1]. Many such ingested foreign bodies pass through the gastrointestinal tract uneventfully [5]. However, in the case of sharp objects such as toothpicks, serious complications can be unavoidable. Ingested toothpicks tend to stick in places where there is natural narrowing, sharp angulations, or congenital gastrointestinal malformation [6]. Li *et al*. in his systemic review of 57 cases found that the duodenum and sigmoid colon are the commonest sites for perforation. Patients diagnosed with perforation of the gastrointestinal tract due to toothpick ingestion are usually men (88%) who present with abdominal pain (70%) or gastrointestinal bleeding (7%). Only 12% of patients had any recollection of swallowing a toothpick. In patients who remembered the event, the onset of symptoms ranged from less than a day to 15 years. The duration of symptoms before diagnosis ranged from one day to nine months [2]. Toothpick ingestion may cause severe, sometimes fatal, internal injuries due to gastrointestinal perforation and migration to adjacent structures [2,4,7]. Diagnosis of toothpick injury can be quite difficult as patients frequently have vague symptoms with no specific physical findings [2,3,6]. Imaging studies are often of limited value as wooden toothpicks are radiolucent in plain films. However, ultrasonography and computed tomography (CT) have been recommended as useful tools for the detection of these foreign bodies, which are often hyperechoic on ultrasonography and of high density on CT [8,9]. Most of the time, the final diagnosis can be achieved through endoscopy, laparoscopy, or laparotomy [4,10]. However, many patients are completely asymptomatic, and objects such as toothpicks may only be uncovered accidentally during other surgical procedures [11].

What is particular about this case is that the foreign body was only discovered at the time of surgery. Interestingly, after surgery, our patient was able to recall the event of toothpick ingestion; however, she did not recall any significant symptoms around the time of the event. A retrospective review of her pre-operative abdominal ultrasonography and MRCP images did not reveal any missed evidence of this foreign body. This could be explained by the fact that there was no inflammatory reaction surrounding the toothpick, which would have raised suspicion of this finding. Very slow migration of the toothpick may probably explain the absence of symptoms in this case [11]. A high index of suspicion of foreign body ingestion should be considered during the assessment of upper abdominal pain of recent onset [2,6,12]. In addition, it is important to exclude any related or missed injury to the adjacent structures when these sharp objects are encountered accidentally during surgery.

## Conclusion

Toothpick ingestion is not an uncommon event and could predispose a patient to serious complications. A high index of suspicion of foreign body ingestion should be considered during the assessment of upper abdominal pain of recent onset. In this particular case, the toothpick was only discovered at the time of surgery; Therefore, it was important during surgery to exclude any related or missed injury to the adjacent structures by this sharp object.

## Consent

Written informed consent was obtained from the patient for publication of this case report and any accompanying images. A copy of the written consent is available for review by the Editor-in-Chief of this journal.

## Competing interests

The authors declare that they have no competing interests.

## Authors' contributions

WA, FR an SYI were major contributors to writing the manuscript. SYI performed the procedure. All authors read and approved the final manuscript.
